# Evaluation of acute flaccid paralysis surveillance indicators in Sokoto state, Nigeria, 2012–2019: a secondary data analysis

**DOI:** 10.1186/s12889-021-11238-1

**Published:** 2021-06-15

**Authors:** Ismail Abdullateef Raji, Auwal Usman Abubakar, Abdulrahman Ahmad, Saheed Gidado, Abdulhakeem Abayomi Olorukooba, Bola Biliaminu Lawal, Chukwuma David Umeokonkwo, Muhammad Balogun

**Affiliations:** 1 Nigeria Field Epidemiology and Laboratory Training Program, Abuja, Nigeria; 2 Department of Community Medicine, Usmanu Danfodiyo University Teaching Hospital, Sokoto, Nigeria; 3 Department of Public Health, Ministry of Health, Sokoto, Nigeria; 4 National Stop Transmission of Polio, Abuja, Nigeria; 5 Department of Community Medicine, Ahmadu Bello University, Zaria, Nigeria; 6 Department of Community Medicine, Alex Ekwueme Federal University Teaching Hospital, Abakaliki, Ebonyi Nigeria

**Keywords:** AFP, Poliomyelitis, Evaluation, Surveillance, Indicators, Sokoto, Nigeria

## Abstract

**Background:**

Nigeria, the last endemic country in the WHO African Region, was certified free of Wild Polio Virus (WPV) in 2020. However, due to low immunity in some communities in Sokoto, outbreaks of the circulating Vaccine Derived Polio Virus (cVDPV) occur. The aim of this study is to evaluate the Acute Flaccid Paralysis (AFP) surveillance indicators in Sokoto state, Nigeria.

**Methods:**

This retrospective study was an analysis of routinely collected AFP surveillance data between 2012 and 2019 by the Sokoto state surveillance network. We assessed the Sokoto state AFP surveillance system using the AFP surveillance performance indicators. We performed all analyses using Microsoft Excel 2019.

**Results:**

Cumulatively, 3001 Acute Flaccid Paralysis (AFP) cases were reported over the evaluation period, out of which 1692 (56.4%) were males, and 2478 (82.4%) were below five years. More than half, 1773 (59.1%), had a fever at the beginning of the disease, and 1911 (63.7%) had asymmetric paralysis. The non-polio AFP rate (9.1 to 23.5% per 100,000 children < 15 years old) and stool adequacy rate (92.5 to 100%) indicate high sensitivity. The proportion of cases that had stool samples collected early, timely transported to the laboratory and arrived at the laboratory in optimal condition were all above the World Health Organization (WHO) minimum standard of 80%. There was inadequate profile documentation of some suspected cases.

**Conclusions:**

Sokoto State has exceeded the WHO minimum standards in most of the AFP surveillance indicators. The performance of the system is sufficient enough to detect any reintroduction of WPV into the state. However, there is a need for improvement in data quality.

## Background

Wild Polio Virus (WPV) is a highly contagious viral disease that leads to poliomyelitis which mainly affects children below five years [1]. It transmitted human to human, mainly via the orofaecal route [2]. Out of 200 cases of WPV serotype 1 (WPV1) infection, one would lead to irreversible floppiness of a limb (usually the lower extremities); and WPV2 and WPV3 have lower ratios of about 1 in 2000 and 1 in 1000, respectively [1, 3] . Among those paralyzed, up to 10% die due to the respiratory muscles’ failure [1].

Following the Global Polio Eradication Initiative’s introduction in 1988, the proportion of new cases of paralysis due to WPV infection has dropped to less than 1 % [1, 4]. The polio searchlight of the world is now on Pakistan and Afghanistan, as Nigeria was recently declared free of WPV [5]. Eliminating the WPV in the remaining endemic countries will lead to the biggest-ever internationally-coordinated open wellbeing exertion in history [1].

Poliomyelitis is a disease targeted for eradication because humans are the only reservoir, and those infected with polio can only transmit the virus for a limited amount of time. Furthermore, the virus survives poorly in the environment and immunization with cheap and effective vaccines interrupts the virus transmission by generating herd immunity [1, 6]. To achieve polio eradication, a country must maintain a sustained vaccine coverage, and every child must receive adequate immunization. The vaccination should include those living in remote and underserved areas and in conflict zones like Borno state in Nigeria (where the last case in Nigeria was reported in 2016) [1]. The presence of just one child with poliovirus puts children worldwide at risk of contracting the virus. The continuous endemicity of poliovirus in certain countries may result in as numerous as 200 thousand new WPV cases each year and, if unchecked, may affect every continent within ten years. Worse of all, once poliovirus causes paralysis, there is no cure [7].

Acute Flaccid Paralysis (AFP) cases present with similar symptoms and signs with poliomyelitis; hence, AFP surveillance is used worldwide to monitor and evaluate the polio eradication initiative [8]. Sensitive AFP surveillance can detect all cases of poliomyelitis for immediate public health action. In regions that have been certified polio-free, effective AFP surveillance is a strategy to continually evaluate the absence of transmission [9, 10]. Therefore, a high-quality AFP surveillance system is needed in proving and maintain the successful interruption of WPV [11].

Nigeria, the last endemic country in the WHO African Region, was certified free of Wild Polio Virus (WPV) in 2020 [5].. However, the circulating Vaccine Derived Polio Virus (cVDPV) affects communities in Africa that are under-immunized, especially among hard to reach communities, migrant populations, and those in conflict zones [12]. This is the case in Sokoto state where in the last year, the state has recorded 6 cases of cVDPV2 from different sources – an indication of low population immunity and favourable factors for the transmission of cVPDVs [13].

The greatest threat to the successes achieved in the interruption of WPV transmission in Nigeria is the rising insecurity and banditry in Northwestern Nigeria. Sokoto state has been facing rising insecurity recently. The insecurity has led to some partially covered areas during Supplementary Immunization Activities (SIAs) and vaccination responses to outbreaks of cVDPV. Overtime time, continuously missing eligible children during vaccination campaigns can lead to a decrease in the community’s immunity, which can lead to outbreaks of cVDPV. Furthermore, surveillance officers’ access to the communities could be limited due to fear of kidnapping and banditry, consequently leading to missing any importation of WPV. Therefore, it is crucial to evaluate the AFP surveillance system to ensure that the state is meeting the minimum required standard for AFP surveillance.

Therefore, it is essential to continuously ensure that the AFP surveillance in Sokoto state is reliable enough to guide public health response towards sustaining the eradication of WPV and stopping cVDPV outbreaks in the context of the polio endgame strategic plan 2019 to 2023 [14].

The aim of this study is to describe the findings from an eight-year AFP surveillance in Sokoto State and assesses the performance of the system with respect to the World Health Organization (WHO) surveillance indicators besides identifying the aspects that need improvement.

## Methods

### Study setting and design

Sokoto state is located in Northwest Nigeria, covering about 27,825 km^2^ [15]. It shares a border with the Niger Republic to the North – making it prone to cross border importation of poliovirus [16], Zamfara state to the south and east, and Kebbi state to the west and south. The state has 23 Local Government Areas (LGAs), out of which four are metropolitan. The projected population of Sokoto state for the year 2019 using a growth rate of 3.01% from the 2006 national census [15] was 5,475,895, with children under-five and under-15 years having a projected population of 114,069 and 268,1364 respectively. The state has a substantial nomadic population, a polio high-risk group [16].

### Description of the AFP surveillance system in Sokoto state

**Fig. 1:**
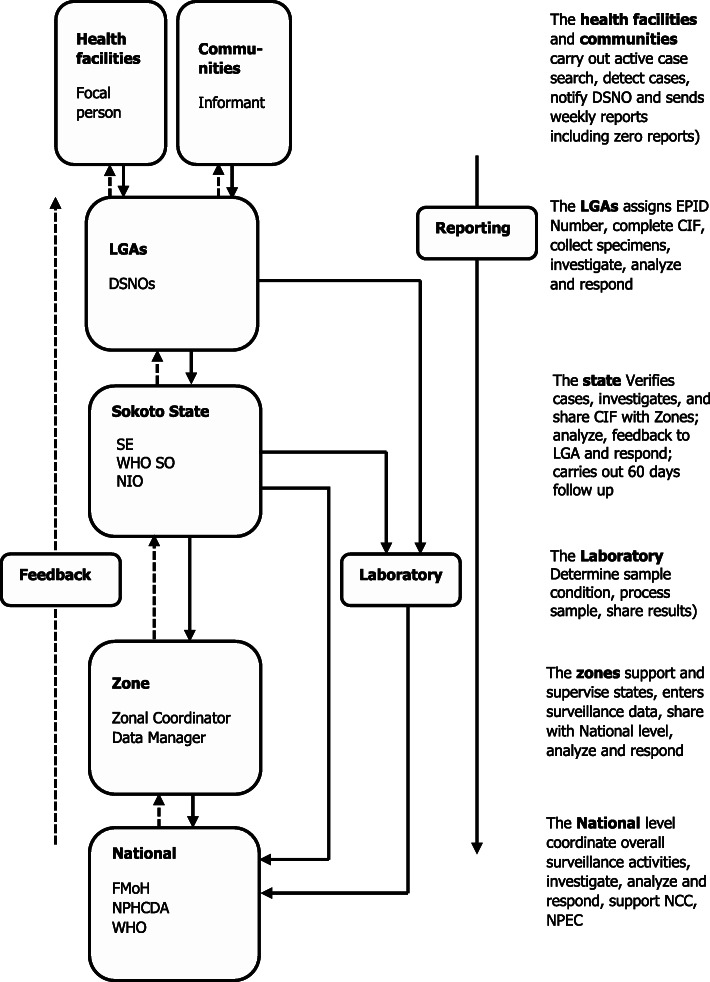
Flow chart of AFP surveillance system. CIF - Case Investigation Form, DSNO - Disease Surveillance and Notification Officer, FMoH - Federal Ministry of Health, NPHCDA - National Primary Health Care Development Agency, NIO - National Immunization Officer, NPEC: National Polio Expert Review Committee, SE: State Epidemiologist, WHO SO - World Health Organization Surveillance Officer

The mode of operation of the Sokoto state AFP surveillance system is similar to the other 35 states and the Federal Capital Territory, and it is part of the broader AFP surveillance system in Nigeria (Fig. 1). In the surveillance system, an AFP case is defined as “Any child under 15 years of age with the acute (sudden) onset of weakness or floppiness of one or more limbs or any person of any age with paralytic illness in whom a clinician suspects poliomyelitis” [17].

The primary purpose of the AFP surveillance system in Sokoto state is to detect and document the presence or absence of WPV. The objectives of the AFP surveillance system in Sokoto state include:

1. To provide data-driven evidence that guides the advancement of strategies that lead to polio eradication.
2. To identify areas of cVDPV in Sokoto state.
3. To investigate all detected AFP cases and demonstrate the non-transmission of WPV in Sokoto state.
4. To assess the effect of Routine Immunization Activities (RIAs) and Supplementary Immunization Activities (SIAs) against polio.

The flow of data in the AFP surveillance in Sokoto state begins with the notification of every case by community informants (such as traditional bone setters) or the health facility focal persons to their respective LGA Disease Surveillance and Notification Officers (DSNOs). The DSNOs are responsible for ensuring that adequate stool samples are collected from suspected cases. They are also responsible for ensuring that the samples get to the national polio laboratory in Ibadan, Oyo state in optimal temperature accompanied with correctly filled AFP Case Investigation Forms (CIFs). The LGA DSNO is responsible for giving regular feedback to the reporting facilities, focal persons and communities.

The state epidemiologist and the state DSNO facilitate and oversee the activities of the LGA DSNO. The WHO cluster consultants assigned to different LGAs are responsible for verifying the suspected cases and conducting 60-day follow-up for all AFP cases. The state epidemiologist gives feedback to the LGAs and reports to the Nigeria Center for Disease Control (NCDC), National Primary Health Care Development Agency (NPHCDA), Federal Ministry of Health (FMoH); and the state DSNO gives feedback to the LGAs on laboratory results (Fig. 1).

The funding of the AFP surveillance System in Sokoto State is by the federal government, Sokoto state government, and LGAs with robust technical and financial support by development partners notably, WHO and Centers for Disease Control and Prevention (CDC) / Africa Field Epidemiology Network (AFENET). WHO sponsors the active case finding visits by DSNOs to AFP reporting sites. Additionally, the organization supports the DSNOs with transportation allowances to attend the monthly surveillance meetings at the states’ capital. CDC/AFENET provides technical and human resource support to all the LGAs to complement Government resources.

### Study design

We conducted a retrospective descriptive analyses of AFP surveillance data in Sokoto state from January 2012 to December 2019. We included AFP cases reported in all the LGAs within the period in this study. We evaluated the AFP performance using the WHO indicators for assessing the AFP surveillance system [18].

### Data collection and analysis

We sieved out information on age, sex, Oral Polio vaccine (OPV) doses, fever at the onset, asymmetry of paralysis, the progression of paralysis in 3 days and classification as true AFP. All analyses were done using Microsoft Excel version 2019. We conducted descriptive analyses and generated core performance indicators and other indicators recommended by the WHO for assessing the AFP surveillance system [18].

## Results

**Fig. 2:**
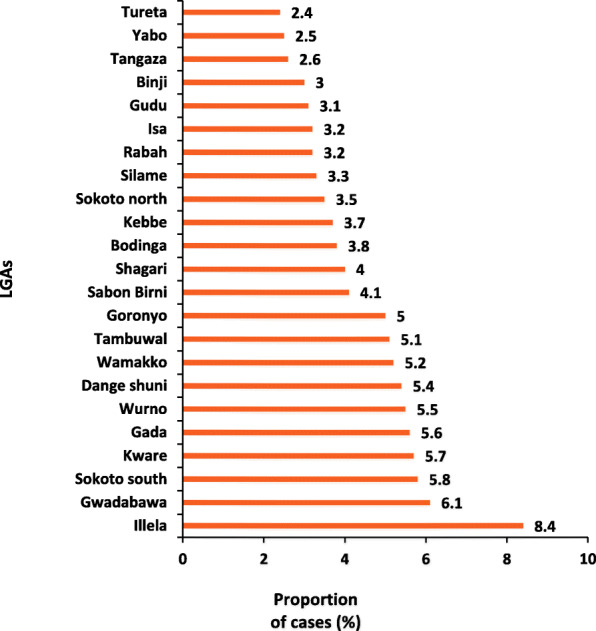
Proportion of cases seen in the LGAs in Sokoto state, 2012–2019

In total, 3001 AFP cases were identified and reported by the AFP surveillance system in Sokoto between January 2012 and December 2019. Out of these, more than half, 1692 (56.4) were males, and more than three-quarters, 2478 (82.4%) were less than five years old. Almost all, 2959 (98.6%) had taken three or more OPV doses. More than half, 1773 (59.1%), had a fever at the onset of the disease, and many 1911 (63.7%) had asymmetric paralysis. More than one-third, 1178 (39.3%) of cases had a progression of paralysis in 3 days and the true AFP rate for the period was 1187 (39.6%) (Table 1). Illela LGA had the highest proportion of cases, 251 (8.4%), while Tureta LGA had the least number of cases, 72 (2.4%), over the evaluation period (Fig. 2).

**Table 1:** Profile of the AFP cases reported in Sokoto state, Nigeria, 2012–2019

Profile	Number of AFP cases	Percent
	(***n*** = 3001)	
**Age group (years)**		
≤ 5	2478	82.5
6–10	383	12.8
11–15	17	0.6
> 15	91	3.0
Unknown age status	32	1.1
**Sex**		
Male	1692	56.4
Female	1309	43.6
**OPV doses**		
< 3	42	1.4
≥ 3	2959	98.6
**Fever at onset**		
Yes	1773	59.1
No	78	2.6
Missing	1150	38.3
**Asymmetry**		
Yes	1911	63.7
No	415	18.3
Missing	541	18.0
**Progression in 3 days**		
Yes	1178	39.3
No	52	1.7
Missing	1771	59.0
**Classified as true AFP**		
Yes	1187	39.6
No	31	1.0
Missing	1783	59.4

**Fig. 3:**
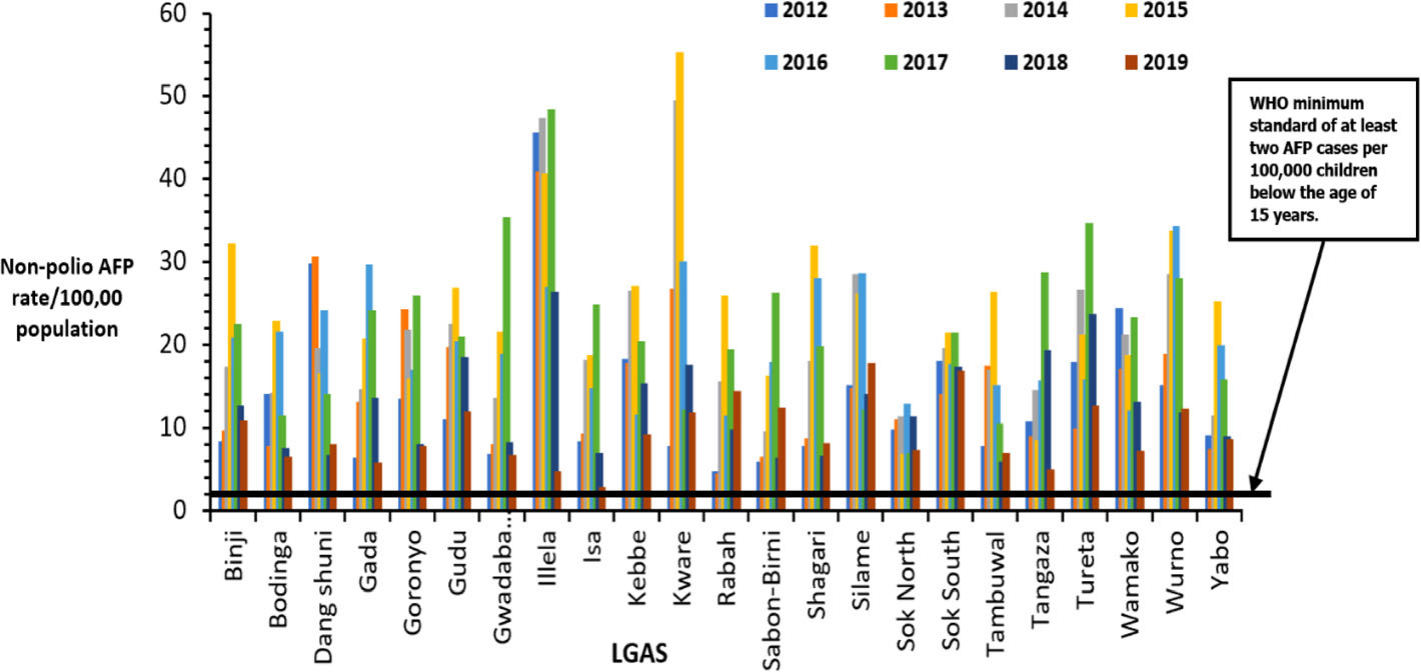
Annualised non-polio AFP rate by LGA for each year in Sokoto state, 2012–2019

Cumulatively, the Sokoto State annualized non-polio AFP detection rate was 16.7 AFP cases per 100,0000 population below 15 years, indicating a sensitive AFP surveillance. Over the evaluation period, the annualized non-polio AFP rate was consistently above the minimum target of ≥2/100,000 in the state. (Table 2). Disaggregating the state cumulative non-polio AFP rate by LGAs showed that all the LGAs consistently surpassed the WHO minimum of 2 AFP cases per 100,000 population of children below 15 years during the eight years evaluation period (Fig. 3).

**Table 2:** AFP performance indicators for Sokoto State, Nigeria, 2012–2019

Performance indicator	Target	State performance							
		2012	2013	2014	2015	2016	2017	2018	2019
Annualized non-polio AFP rate /100,000 < 15 years population	≥3	13.5	15	20.1	23.5	19.1	21.7	11.8	9.1
Proportion of AFP cases with two adequate stool specimens	≥80%	92.5	97	99.3	100	100	99.7	96.7	96.1
Timeliness of monthly reporting	≥80%	100	100	89	100	96	100	100	96
completeness of monthly reporting	≥90%	100	100	99	100	97	100	100	97
Proportion of AFP cases investigated within 48 h of notification	≥80%	98.3	100	100	99.4	100	100	100	100
Reported AFP cases with follow-up exam at least 60 days after paralysis onset.	≥80%	80.8	100	100	100	100	100	100	100
The proportion of specimens that arrived at a WHO accredited laboratory < 3 days of being sent	≥80%	100	100	100	100	100	100	100	100
Proportion of stool specimens arriving at the laboratory in good condition	≥80%	99.3	99.5	100	99.8	99	99	100	100
Proportion of stool specimens from which Non-Polio Enterovirus (NPENT) was isolated	≥10%	14.9	11.1	11.4	9.5	14	13	14	13
AFP surveillance index	≥1.6	12.5	14.6	20.0	23.5	19.1	21.6	11.4	8.7

**Fig. 4:**
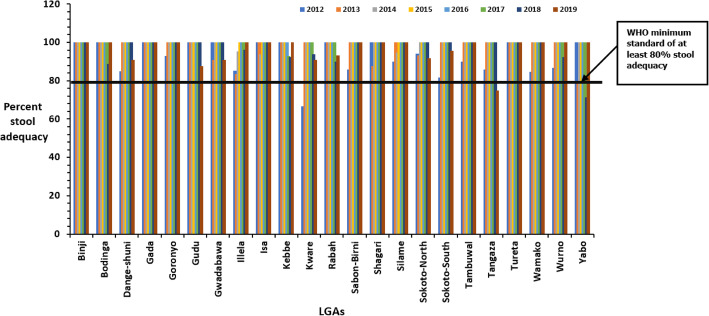
Stool adequacy rate by LGA for each year in Sokoto state, 2012–2019

There was a consistent increase in the proportion of AFP cases with adequate stools from 92.5% in 2012 to 100% in 2016 and a drop to 96% in 2018 and 2019. Over the evaluation period, the stool adequacy performance was above the minimum target of 80% for the state (Table 2). Disaggregated by LGAs, all the LGAs met the minimum standard except, Kware, 67% in 2012; Yabo, 71% in 2018 and Tangaza, 75% in 2019 (Fig. 4).

All stool samples arrived at the laboratory within 72 h of being sent during the evaluation period (Table 2). The laboratory performance indicator, Non-polio Enterovirus (NPENT) rate, was above the minimum level required over the 8-year evaluation period. (Table 2).

## Discussion

This study involved a state-wide analysis of AFP surveillance data in Sokoto State and reports the findings of the evaluation of APF surveillance indicators from 2012 to 2019. Over the evaluation period, we found that children below five years were most affected, with over 80% of the cases. This finding corroborates what has been stated by WHO that under-five children are most affected [19]. This finding’s significance is that poliomyelitis mainly affects children below five years [7]; therefore, if the AFP surveillance is picking children of this age group, then any WPV or cVDPV case will be easily detected. The finding in this study is comparable to what was reported in the evaluation of surveillance system in Ibadan (74.3%) [20] and Akwa Ibom (82.5%) [21] states in Nigeria and Ghana(76.3%) [22]. However, a surveillance system evaluation in Zambia showed that 63% of cases were in the age group 10–15 years [23]; although a significant proportion of the cases did not have their age documented in the study, which could have been responsible for their finding. More than half (56.7%) of the AFP cases observed in this evaluation were males. This finding is similar to what was reported in Nigeria [24], However, studies in Akwa Ibom [25] and Nasarawa [26] states reported that Females were slightly more than males.

Almost all the AFP cases have had at least three doses of OPV through RIAs and SIAs activities. This is encouraging because it is an indication that Sokoto State is implementing the global polio eradication strategies. Worthy of note in the surveillance data was that some characteristics of the AFP cases were not documented. For example, 38.3% of the cases did not have documentation on the presence of fever at the onset of paralysis, and 59% did not have documentation on the progression of paralysis. This information is essential in determining whether a case is a “hot” case or not [17, 27]. A hot case is an AFP case that is very likely to be WPV. To determine a hot case, the patient must have three of five criteria: less than five years old, clinically compatible (Asymmetric paralysis, fever at onset and rapid progression of paralysis), vaccination status less than 3 OPV doses, member of a high-risk group (such as migrants, nomads and security compromised area), occurrence in a polio-free state [17]. Therefore, this poor quality of data can affect the performance of the surveillance system.

Maintaining a sensitive surveillance system that can detect WPV is critical in eradicating poliomyelitis by enabling early response to importations and certifying the complete interruption of transmission [14]. A well-performing AFP surveillance system should pick a minimum of two AFP cases per 100,00 children younger than 15 years [28]. This is used as a proxy of the sensitivity of AFP surveillance system. This indicator measures the surveillance system’s capacity to detect AFP cases due to other causes than WPV [17]. In this study, we found the AFP surveillance system in Sokoto to be sensitive. The minimum standard for non-polio AFP rate was surpassed throughout the period under review. It is crucial to monitor LGA performance because state-level indicators may mask wide variation in LGA performance [17]; therefore, we disaggregated the data by LGA. We found that all LGAs performed above the minimum standard. This finding is encouraging, indicating that no LGA is hiding behind the state’s overall success. The sensitive AFP surveillance system is likely to pick any reintroduction of WPV or an outbreak of cVDPV in all the LGAs. A similar finding was reported in Kaduna state [29].

A stool specimen is adequate if collected 24–48 h apart and within two weeks of the onset of paralysis and arriving at the laboratory in good condition [28]. The results from this surveillance evaluation showed that the proportion of stool samples adequately collected throughout the evaluation period was consistently above the minimum standard of 80%. After disaggregating, all LGAs performed well, except 3 LGAs (Kware in 2012, Yabo in 2018 and Tangaza in 2019). This finding could be an indication that the community and parents are aware of the AFP surveillance system, leading to early detection and reporting. The finding could also be an indication that there are minimal causes of delays such as lack of involvement of health workers or inadequate logistics such as stock-out of kit sand transport.

The non-polio AFP rate and the stool adequacy rate are used as the standard for assessing the quality of AFP surveillance [27]. These two indicators can be combined into a single indicator of AFP surveillance quality, the surveillance index, which can be used to compare progress over time and or geographic differences [17, 27]. In this surveillance evaluation, the surveillance index for the state was greater than 2.5, indicating a robust AFP surveillance on average [17]. Using this index in maps helps in identifying areas of risk. Fortunately, in Sokoto State, no area of risk was identified using this index. The success in the surveillance index could be due to the regular capacity building and financial support provided by the WHO to the LGA DSNOs.

In addition to finding AFP cases, timeliness, and the quality of investigation of suspected cases are also vital in achieving an AFP surveillance system’s objectives. Almost all the suspected cases were investigated within 48 h of notification during the evaluation period. This could be attributed to proper training and supervision from WHO cluster consultants in the various LGAs in the state.

Laboratory investigation is fundamental to the confirmation of WPV; therefore, the integrity of the faecal samples arriving in the laboratory should be good enough for laboratory confirmation of the presence or absence of the virus with a reasonable level of certainty. No WPV was isolated in Sokoto State during the evaluation period; therefore, with the high stool adequacy rate, any form of poliovirus transmission will be most likely picked by the AFP surveillance in Sokoto state. This study’s finding is similar to what was reported in the evaluation done in Oyo state, Southern Nigeria, where stool adequacy was above the WHO recommended standard of 80% [30].

To maximize the opportunity to isolate the poliovirus, with the highest probability occurring within the first 14 days, some AFP surveillance indicators assess the timeliness of certain surveillance activities [27]. A minimum of 80% of faecal samples should reach the laboratory within three days of sample collection [28]. The result from this evaluation revealed that in Sokoto State, all the stool samples were received in the laboratory within three days during the evaluation period. In Nigeria, Sokoto State inclusive, the LGA DSNOs are responsible for the samples’ immediate transportation to the laboratory. This study’s positive finding on the timeliness of transportation of samples to the laboratory indicates that the LGA DSNOs were efficient over the evaluation period to ensure that the samples reach the laboratory in good time.

The arrival of the stool samples in the laboratory within three days and samples being in good condition is pivotal in the detection of poliovirus. These indicators also assess the timeliness of surveillance activities [27]. In this evaluation, at least 99% of samples reached the designated laboratory in perfect condition, and all samples arrived at the laboratory within three days. This finding gives a high degree of confidence that whatever findings in the laboratory reflect the actual situation. This positive finding could be attributed to the frequent sponsored training on polio surveillance activities by WHO in the state, close monitoring of surveillance activities by cluster consultants and provision of stipends for surveillance officers. The AFP surveillance system met the target for timeliness of monthly reporting over the evaluation period. This finding is important because this allows the state to take all necessary early actions to ensure polio certification.

The NPENT rate assesses how the AFP surveillance system can maintain the reverse cold chain. It also assesses the performance of the laboratories in the routine isolation of enteroviruses [17]. Sokoto state has performed well in this indicator over the evaluation period by exceeding the minimum value of 10%. This finding is important, especially in the post-polio era in Sokoto state, which still reports cVDPVs. It has been established that cVDPVs have the potential to combine and recombine with other enteroviruses, which can give rise to new pathogenic strains [31]. Therefore, adequate NPENT surveillance will help detect and control any outbreak. Similar NPENT rates were recorded in the neighbouring Kebbi state between 2010 and 2015 [32].

Overall, the AFP surveillance indicators are meeting up with the minimum targets. However, this should not create a state of complacency as it was observed in Jigawa state in 2011, where all the minimum standard for certification were surpassed. Still, following analyses of environmental samples, WPV and cVDPV were detected, indicating that certain chains of transmission had been missed [27]. Therefore, surpassing most of the surveillance indicator targets should not allow the lowering of surveillance guards.

One of the limitations observed in this study is incomplete data entry. Some information on the clinical history and immunization history was missing in the database, we analysed the available data after efforts were made to triangulate the data from other sources. Another area of deficiency observed in this paper is that the surveillance data obtained had no information on the timeliness of specimen processing. Therefore, we could not assess if the laboratory sends back stool sample results within 28 days of receipt of samples in the laboratory. This finding perhaps points to a lack of laboratory results dissemination through the feedback channel or lack of entry of data into the database. Therefore, there is a need for surveillance officers to follow up on the results of stool samples they have submitted to the polio isolation laboratory and ensure that findings are entered into the local database in Sokoto state.

## Conclusion

The AFP Surveillance system in Sokoto State has performed well over the past eight years by exceeding most of the minimum WHO targets both at the state and LGA levels. The system is sensitive enough to detect any outbreak of wild or circulating polio virus. However, there is inadequate documentation of laboratory results and some profile information on the suspected cases. We recommend that the state ministry of health organize training on data management and supportive supervision for data managers and modify the database to include logical check functions to minimize the challenge of missing data.

## Data Availability

Public access to the datasets analyzed in this study is closed. However, the data is available from the corresponding author on request.

## Abbreviations

AFENET: Africa Field Epidemiology Network
AFP: Acute Flaccid Paralysis
CDC: Centres for Disease Control and Prevention
CIFs: Case Investigation Forms
cVDPV: circulating Vaccine Derived Polio Virus
DSNOs: Disease Surveillance and Notification Officers
FMoH: Federal Ministry of Health
LGAs: Local Government Areas
NCDC: Nigeria Center for Disease Control
NPENT: Non-polio Enterovirus
NPHCDA: National Primary Health Care Development Agency
OPV: Oral Polio vaccine
RIAs: Routine Immunization Activities
SIAs: Supplementary Immunization Activities
WPV: Wild Polio Virus
